# Cancer Stem Cells Persist Despite Cellular Damage, Emergence of the Refractory Cell Population

**DOI:** 10.1245/s10434-023-13849-x

**Published:** 2023-07-31

**Authors:** Ayumi Nagae, Norikatsu Miyoshi, Shiki Fujino, Masafumi Horie, Shinichi Yachida, Masaru Sasaki, Yuki Sekido, Tsuyoshi Hata, Atsushi Hamabe, Takayuki Ogino, Hidekazu Takahashi, Mamoru Uemura, Hirofumi Yamamoto, Yuichiro Doki, Hidetoshi Eguchi

**Affiliations:** 1 Department of Gastroenterological Surgery, Osaka University Graduate School of Medicine, Suita, Osaka Japan; 2 Department of Innovative Oncology Research and Regenerative Medicine, Osaka International Cancer Institute, Osaka, Japan; 3 Department of Molecular and Cellular Pathology, Graduate School of Medical Sciences, Kanazawa University, Kanazawa, Japan; 4 Department of Cancer Genome Informatics, Osaka University Graduate School of Medicine, Suita-City, Osaka, Japan; 5 Division of Pediatric Gastroenterology, Hepatology, and Nutrition, The Children’s Hospital of Philadelphia, Philadelphia, PA USA

**Keywords:** Cancer stem cells, Anticancer treatment, Organoids, Xenograft model, Colorectal cancer

## Abstract

**Purpose:**

Cancer stem cells (CSCs) are responsible for chemotherapy resistance and have unique properties that protect them from chemotherapy. Investigating CSCs may help to identify the population that is more resistant to treatments, leading to recurrence. We evaluated persisting CSCs, emerging after chemotherapy that cause tumor recurrence.

**Methods:**

Using human colorectal cancer organoids prepared from surgical specimens, we looked at changes in CSCs, the emergence and changes in the original population, which single-cell analysis identified.

**Results:**

With regards to changes in cancer stem cell markers, CD44 showed low levels after 5-fluorouracil administration. Once the CD44-ve population was sorted and cultured, the CD44+ve population gradually emerged, and the CD44-ve population decreased. Compared with the CD44-ve population of an organoid parent, the CD44-ve population proliferated after chemotherapeutic agent stimulation. The CD44-ve population was derived from the CD44+ve population before chemotherapeutic agents. In addition, when the CD44 variants were evaluated, the CD44v9 population remained. In single-cell analysis, we found that POU5F1 was highly expressed in the CD44low population. Velocity analysis showed that the CD44-ve population was induced after chemotherapy and expressed POU5F1. POU5F1-EGFP-Casp9 transfected organoids resulted in the appearance of a CD44-ve population after administration of a chemotherapeutic reagent. Both in vivo and in vitro, the dimerizer administration inhibited tumor growth significantly.

**Conclusions:**

POU5F1 is involved in chemotherapy resistance in relation to stemness. For the treatment against refractory tumors, such as the recurrence after chemotherapy, the treatment should target the emerging specific population such as CD44 (or CD44v9) and proliferative cancer cells.

**Supplementary Information:**

The online version contains supplementary material available at 10.1245/s10434-023-13849-x.

Colorectal cancer (CRC) is the third most common type of newly diagnosed cancer and the second leading cause of cancer-related deaths worldwide.^1^ Patients with early-stage CRC are primarily treated surgically, whereas those with advanced CRC require additional perioperative radiation therapy and chemotherapy.^2^ Although radiation therapy and chemotherapy may be curative in a number of cancer types, success is limited by the development of resistance. Cancer stem cells (CSCs) are one cause of this problem. CSCs were first noticed in acute myeloid leukemia.^3^ The de-differentiation and transformation of normal cancer cells into stem cell-like cells may be the mechanism that induces the development of CSCs.^4,5^

Cells within a tumor have diverse phenotypic systems, and therapeutic resistance is implicated in this diversity.^6,7^ CSCs contribute to this diversity. Conventional chemotherapy targets non-CSCs in the tumor and fails to eliminate CSCs, resulting in limited efficacy.^8–10^ This is evidenced by CSCs being more resistant to conventional therapies than non-CSCs.^11,12^ Even treatments that completely eliminate non-CSCs may be able to repopulate tumors if only CSCs remain.^7,13^

CD44 has been proposed as an important cancer stem cell marker in several cancers.^14,15^ CD44 is a cell surface glycoprotein that plays roles in the adhesion of the cytoskeleton to the extracellular matrix, cell–cell interactions, and cell migration.^15-17^ CD44 knockdown has been reported to prevent tumor formation and clonogenesis.^18^ The ability of CD44+ve/CD24+ve cells to differentiate into the enterocyte, enteroendocrine, and goblet cell lineages in vitro also has been established.^19^ CD44 overexpression has been linked to high cancer aggressiveness and resistance.^20^

CSCs, with their unique surface markers, have unique properties that protect them against cytotoxic drugs. Therefore, investigations into CSCs may help identify the population that is more resistant to treatments. Identification of resistant CSCs after chemotherapy is very helpful for the treatment of refractory tumors, and the investigation of the surface markers is as well. This study evaluated persisting CSCs (even after stimulation with chemotherapeutic agents) that cause tumor recurrence.

## Materials and Methods

### CRC Cell Line Culture

Human CRC cell lines (DLD-1, HCT116, HT29, RKO, and SW480) were a gift from Dr. Bert Vogelstein (Johns Hopkins University, Baltimore, MD). The cells were incubated in Dulbecco’s modified Eagle’s medium (DMEM; Sigma-Aldrich, St. Louis, MO) supplemented with 10% fetal bovine serum (Thermo Fisher Scientific Inc., Waltham, MA), 1% GlutaMAX-I (Thermo Fisher Scientific Inc.), and 1% penicillin/streptomycin/amphotericin B (Wako Pure Chemical Industries Ltd., Osaka, Japan). The cells were maintained at 37 °C in a humidified atmosphere containing 5% CO_2_.

### Establishment and Culture of Human Organoids

CRC tissue was cut into small pieces, dissociated using 1 mg/mL collagenase (C6885; Sigma-Aldrich, St. Louis, MO) in DMEM, and shaken in a bioshaker BR-13FP (Taitec Co., Saitama, Japan) at 6 × *g* for 15 min at 37 °C. Dissociated tissues were filtered through a custom-made filter (Sansho Co., Tokyo, Japan), centrifuged at 400 × *g* for 5 min at room temperature (RT, 20-25 °C), and the collected cell pellets were resuspended in a culture medium (modified stem cell culture medium^21^). Suspended human organoids (iCC603, iCC821, and iCC724) were seeded onto plates coated with Matrigel (Corning Inc., Corning, NY). The medium was changed every 2–3 days. After the cells had spread to more than 50% of the plate, they were passaged with Accutase (Nacalai Tesque, Kyoto, Japan) for approximately 5 min. Cells were collected, resuspended in the culture medium, and seeded onto Matrigel-coated plates. Obtaining the medical records and clinical samples, written, informed consent was obtained from all participants following the ethics guidelines of the Osaka International Cancer Institute.

### Flow Cytometry

The expression of surface proteins within the collected cells was determined by using flow cytometry (FC). Cells were dissociated with Accutase (Nacalai Tesque) and stained with CD24 (1555427; BD Biosciences), CD44 (103012; BioLegend, 338820; BioLegend), CD44v5 (L MCA1729; Bio-Rad), CD44v6 (MCA1730; Bio-Rad), CD44v7 (MCA1731; Bio-Rad), CD44v9 (LKG-M003; Cosmo Bio), CD133 (372808; BioLegend), and 7-AAD (372808; BD Biosciences). The relative fluorescence intensities were measured by using an SH800 cell sorter (Sony Corporation, Tokyo, Japan). A two-dimensionality reduction step was performed using *t*-distributed stochastic neighbor embedding (t-SNE) to visualize high-dimensional cell surface marker expression data in a low-dimensional space. Data were analyzed by using the FlowJo software, Version 10.2 (FlowJo).

### Time Course Evaluation

For the withdrawal period, we divided human organoids into two groups: one in which chemotherapeutic agents were administered for 3 days, and then, CD44 marker expression was analyzed (=Day 0), and the other in which the medium was changed and CD44 expression was analyzed after 1, 2, and 3 days (=Post 1, 2, and 3 days). For the duration of treatment, human organoids were treated with agents for 1 to 5 days to analyze the expression of CD44 markers.

### RNA Preparation and Quantitative Reverse Transcription-Polymerase Chain Reaction (qRT-PCR)

Gene expression microarrays were analyzed in CD44 cells. CD44+ve and CD44-ve cells were sorted using an SH800 cell sorter (Sony Corporation, Tokyo, Japan).

Total RNA was prepared by using an RNA Purification Kit (Qiagen GmbH, Hilden, Germany). Reverse transcription was performed using a Transcriptor First Standard cDNA Synthesis Kit (Roche Diagnostics, Tokyo, Japan). qRT-PCR was performed by using the FastStart TaqMan Probe Master (Roche Diagnostics), the Universal Probe Library platform (Roche Diagnostics), and the CFX Connect Real-Time PCR Detection System (Bio-Rad Laboratories, Hercules, CA) for cDNA amplification of target genes. The primers and Universal Probe Library probes used in this study are listed in Supplementary Table S1.

### Proliferation Assay

Immediately after FC, 1 × 10^5^ cells of CD44+ve and CD44-ve human organoids were seeded into 12-well plates. Proliferation in the same well was evaluated over time by live-cell imaging using the IncuCyte S3 Live-Cell Analysis System (Sartorius, USA) on adhesion cell fluence.

### Drug Sensitivity Assay

Cell lines (2 × 10^3^ cells/well) and organoids (5 × 10^3^ cells/well) were seeded and cultured in 96-well plates or 6-well plates. When cells were 60–70% confluent, they were treated with 5-FU (0.3–150 μg/mL for cell lines and 0.003–300 μg/mL for primary culture cells). After 3 days, cell viability was measured using the CCK-8 assay (Dojindo Molecular Technologies, Inc.).

### Xenograft Model for Histological Examination of Primary Cultured Cells

Histological examination of parent and CD44-ve cells after chemotherapeutic agents were performed using a xenograft model. Accutase-dissociated cells (5 × 10^5^ cells) suspended in Matrigel (BD Biosciences, Franklin Lakes, NJ) were subcutaneously transplanted into the dorsal flanks of 7-week-old, male, nonobese, diabetes/severe combined immunodeficiency mice (CLEA, Tokyo, Japan). 2D organoids (2DOs) were injected into different mice. The mice were sacrificed 3 weeks after transplantation, or when the tumor diameters were 15 mm, by cervical dislocation under anesthesia. The mice were weighed weekly, and no mice had reduced body weight. Xenograft tumors were fixed in formalin, processed through a series of graded ethanol concentrations, embedded in paraffin, and sectioned. Sections were stained with hematoxylin and eosin. After deparaffinization and blocking, sections of the CRC specimen were incubated with primary anti-POU5F1 rabbit polyclonal antibody (#2570; Cell Signaling Technology Inc., Beverly, MA) and primary anti-Ki-67 rabbit monoclonal antibody (ab16667; Abcam, Cambridge, UK) at a dilution of 1:200 overnight at 4 °C. Vectastain Universal Elite (Vector Laboratories, Burlingame, CA) was used to detect the signal. Diaminobenzidine was used for color modification. All sections were counterstained with hematoxylin. The Osaka International Cancer Institute Review Board and Animal Research Committee approved this study.

### Establishment of DsRed-transfected Cells

The vector pLV[Exp]-Neo-CMV>DsRed_Express2 (Vector Builder, VB900088-2435mhv) was transfected into 2DOs using the Lentiviral High-Titer Packaging Mix with pLVSIN (Takara Bio Inc., Shiga, Japan), according to the procedure described in our previous report.^22^ Subsequently, the DsRed-positive cells were sorted by using an SH800S cell sorter (Sony Corporation, Tokyo, Japan).

### Establishment of POU5F1-EGFP-Casp9 Cells

PL-SIN-Oct4-EGFP, which expresses EGFP under the control of the POU5F1(Oct4) promoter, was a gift from James Ellis (Addgene plasmid # 21319).^23^ In addition, by pMSCV-F-del Casp9.IRES.GFP, kindly gifted by David Spencer (Addgene plasmid # 15567),^24^ we established cells expressing EGFP under the OCT4 (POU5F1) promoter with inducible caspase 9. We digested sequence-encoding caspase 9 with restriction enzymes EcoRI-HF (R3101S; New England Biolabs) and XhoI (R0146S; New England Biolabs, Beverly, MA). The DNA fragment of caspase 9 was extracted from E-Gel CloneWel 0.8% (G6500ST; Thermo Fisher Scientific) using the E-Gel Power Snap Electrophoresis System (Thermo Fisher Scientific).

This fragment was amplified by using CloneAmp HiFi PCR Premix (Z9298N; Takara Bio) with primers (FW_gaattctgcagtcgatcgagggtcaggtgg, RV_ccgcggtaccgtcgacttagtcgagtcgagtcgttagc). Amplification. PL-SIN-Oct4-EGFP was linearized by a restriction enzyme, *Sal*I-HF (R3138S; New England Biolabs). The amplified fragments and linearized vector were used for the cloning reaction by the In-Fusion HD Cloning Kit (Z9648N; Takara Bio). The transformation procedure was performed using Competent High *E. Coli* DH5α (TYB-DNA903; Toyobo, Osaka, Japan), and the plasmid was extracted using the Qiagen Plasmid Plus Midi Kit (12945; Qiagen). The nucleotide sequence of the vector was confirmed by Sanger sequencing performed by GENEWIZ Japan Corp. (Kawaguchi, Japan). Primer extension sequencing was performed using Applied Biosystems BigDye version 3.1, and the reactions were then run on an Applied Biosystem's 3730xl DNA Analyzer. The constructed vector was transfected into two PDOs (iCC603 and iCC724) by using Lentiviral High Titer Packaging Mix with pLVSIN (Takara Bio). EGFP-positive cells were cloned by single-cell sorting using an SH800 cell sorter (Sony Corporation, Tokyo, Japan). *POU5F1* expression was confirmed by PCR, and a decrease in the number of EGFP-positive cells was evaluated by the administration of B/B Homodimerizer (Z5059N; Takara Bio) (dimerizer). The mean provirus copy number was 6.05 (±1.16, *n* = 6), as measured using the Let-X Provirus Quantitation Kit (Z1239N; Takara Bio).

### Single-Cell RNA Sequencing of Human Organoids and Generation of Data Matrix

Single-cell library preparation was performed following the manufacturer’s instructions for the Chromium Next GEM Single Cell 3′ Reagent Kits (v3.1; 10x Genomics, Pleasanton, CA), and the libraries were sequenced on a HiSeq X sequencer (Illumina, San Diego, CA). Cell Ranger pipeline (version 6.1.2) was applied to generate the data matrix. Raw reads were aligned to the human reference genome (GRCh 38) by using STAR aligner. Seurat (version 4.1.0) was used for quality control and downstream analysis. Poor quality cells were filtered out using the following parameter: *nFeature_RNA* 1000 – 7000 and *percent.mt* < 15, and finally, 3,654 cells that passed QC were finally used for further analysis. Uniform manifold approximation and projection (UMAP) visualization was used for dimensionality reduction analysis with the following parameters: resolution 0.5 and perplexity 40. Marker genes discriminating the different clusters were identified by using the *FindAllMarkers* function. To calculate RNA velocity, the velocyto R package (v0.6) was applied.

### Statistical Analysis

Continuous variables were expressed as means ± standard deviations or standard errors of the means. Student’s *t* tests were used to analyze the differences between two independent groups. All statistical analyses were performed by using JMP (SAS Institute Inc., Cary, NC). *P* values < 0.05 were considered statistically significant.

## Results

### Changes in CSC Marker Expression in Human Cell Lines and Organoids

**Fig. 1:**
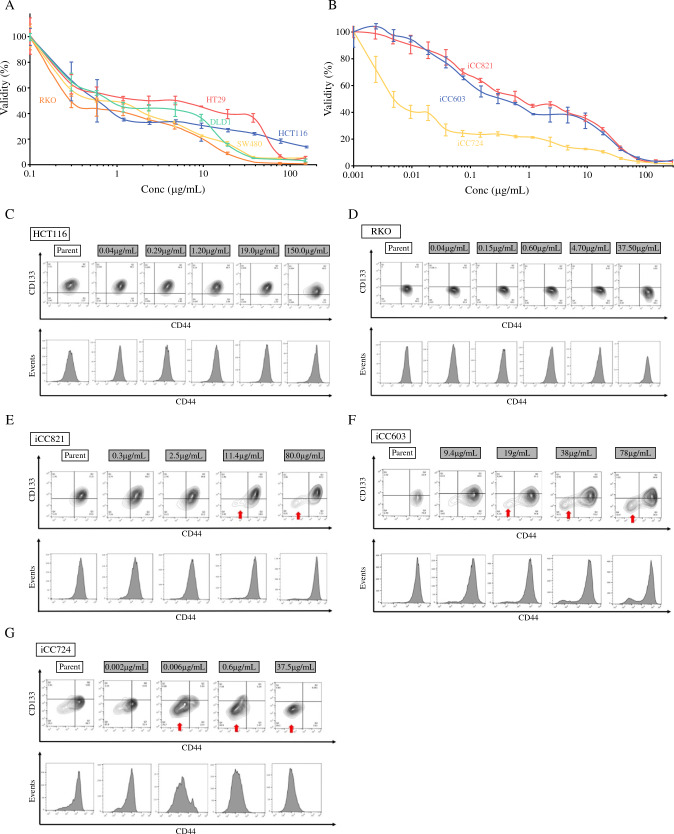
Chemosensitivity assay and flow cytometry in human cell lines and organoids. **A** Drug sensitivity assay using 5-fluorouracil (5-FU) on five CRC cell lines (DLD-1, HCT116, HT29, RKO, and SW480). **B** Drug sensitivity assay using 5-FU on three CRC human organoids (iCC603, iCC821, and iCC724). **C** Changes in CD44 and CD133 expression after 5-FU (150, 19, 1.2, 0.29, 0.04 μg/mL) administration to HCT116. **D** Changes in CD44 and CD133 expression after 5-FU (37.5, 4.7, 0.60, 0.15, 0.04 μg/mL) administration to RKO. **E** Changes in CD44 and CD133 expression after 5-FU (80, 11.4, 2.5, 0.3 μg/mL) administration to iCC821. **F** Changes in CD44 and CD133 expression after 5-FU (78, 38, 19, 9.4 μg/mL) administration to iCC603. **G.** Changes in CD44 and CD133 expression after 5-FU (37.5, 0.6, 0.006, 0.002 μg /mL) administration to iCC721

We performed a chemosensitivity assay by using 5-FU in the cell lines and found that the calculated half-maximal inhibitory concentration (IC_50_) values were 2.40 μg/mL for HT29, 1.21 μg/mL for DLD-1, 0.80 μg/mL for SW480, 0.60 μg/mL for HCT116, and 0.47 μg/mL for RKO. HCT116 and RKO cells were found to be more sensitive to 5-FU (Fig. 1A). For these two cell lines, we administered 5-FU at five different concentrations, with viability ranging from 0% to 100%, and performed FC for CD44 to visualize changes in the cells that would survive. In all cases, only CD44+ve areas remained (Fig. 1C, D). When 5-FU was administered in the same manner in organoids, the IC_50_ values were 0.960 μg/mL for iCC821, 0.523 μg/mL for iCC603, and 0.0062 μg/mL for iCC724 (Fig. 1B). When we examined the changes in cells that survived at different concentrations, a new CD44-ve population emerged as the concentration of 5-FU increased (Figs. 1E–G).

### Time Course and Stem Marker Analysis

**Fig. 2:**
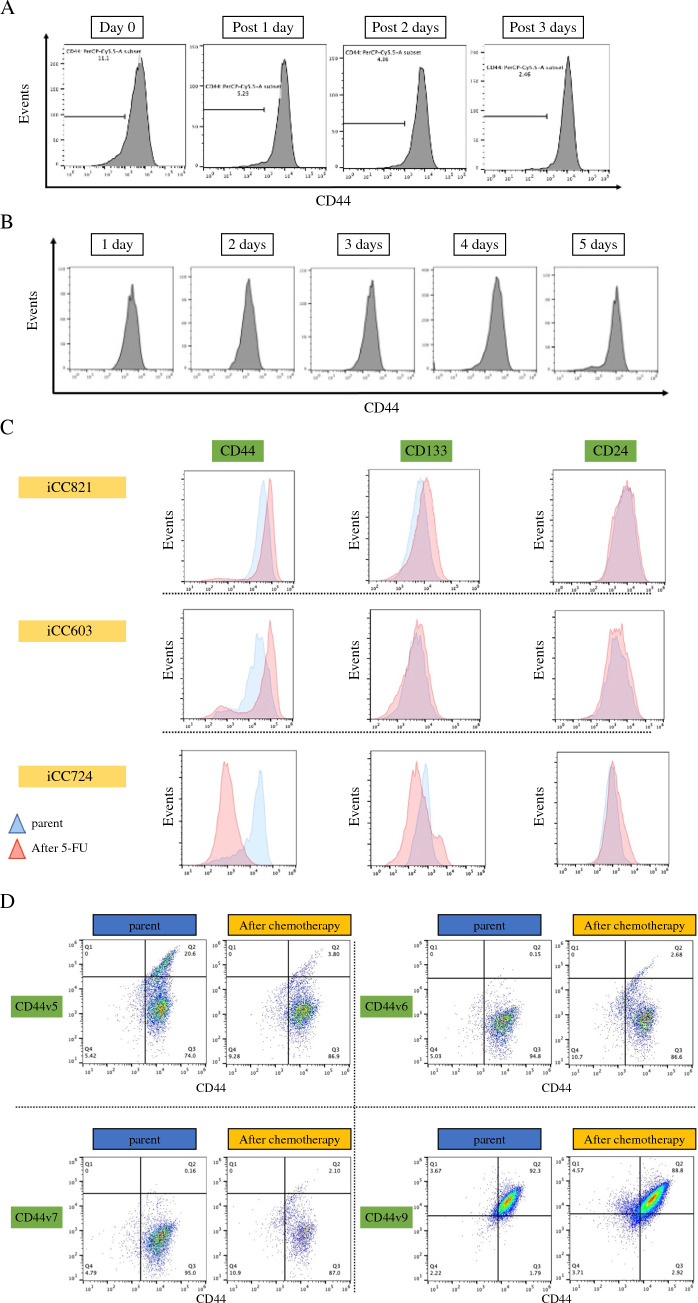
Time course and stem marker analysis. **A** No withdrawal period (=Day 0) has a high percentage of the CD44-ve population. When the withdrawal period is extended, the CD44-ve population decreases. **B** During the administration period, the CD44-ve population began to appear after 3 days of treatment; after 5 days, it was difficult to isolate due to low cell counts. **C** Unlike CD44 expression, CD133 and CD24 expression did not change after 5-fluorouracil (5-FU) administration (iCC821 80 μg/mL, iCC603 38 μg/mL, iCC724 37.5 μg/mL). **D** The CD44 variants were evaluated and these expressions did not change after 5-FU administration (iCC603 38 μg/mL)

First, we examined when the CD44-ve population appeared during the withdrawal/administration periods. During the withdrawal period, the percentage of CD44-ve was higher on Day 0 than on Post 1–3 (Fig. 2A). During the administration period, a CD44-ve population began to appear after 3 days of administration and appeared more clearly after 5 days. After 5 days, the cell count was low and difficult to verify. Therefore, we decided to proceed with the experiment after 3 days of administration (Fig. 2B).

We also examined the changes in cancer stem cell markers. CD133 and CD24 did not show low levels after 5-FU administration, similar to those of CD44 (Fig. 2C). CD44 variants also were evaluated; however, all variants did not show low levels (Fig. 2D).

### Changes in CSC Markers after Chemotherapeutic Agents

**Fig. 3:**
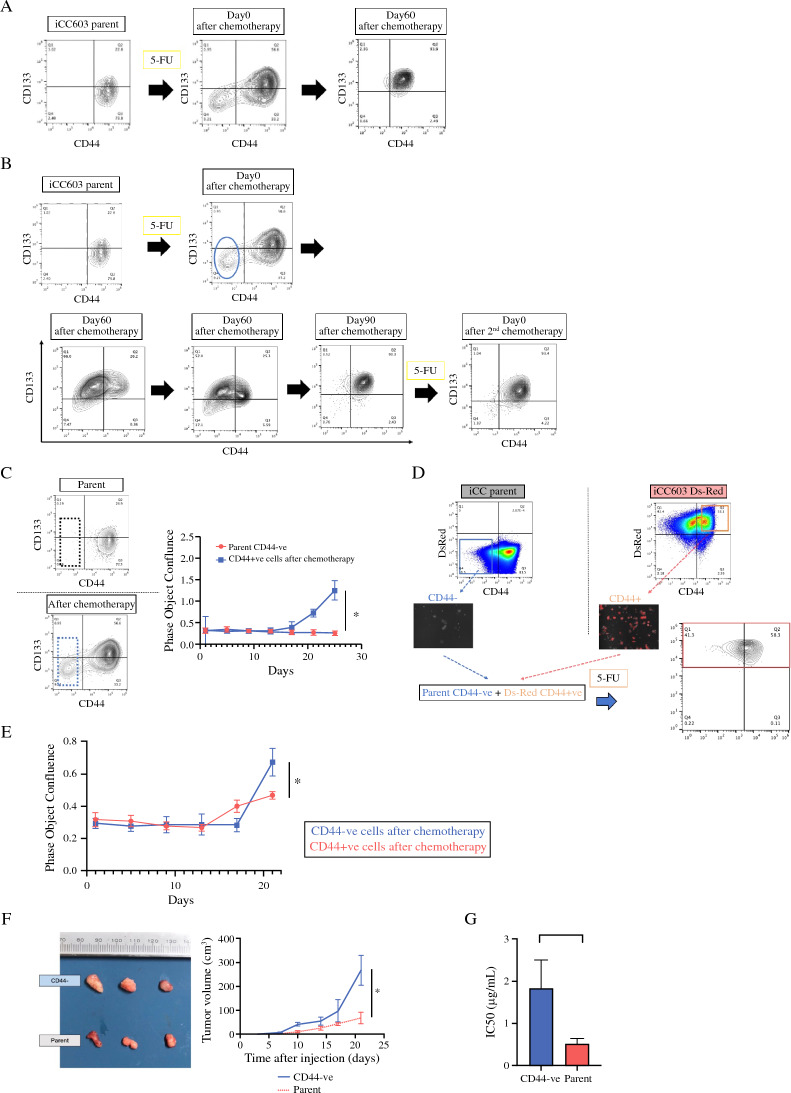
CSC markers after chemotherapy. **A** After 5-fluorouracil (5-FU) (38 μg/mL) was administered to iCC603, CD44-ve and CD44+ve populations appeared. After 60 days, their surface markers reverted to the original population. **B** We can make CD44+ve population out of CD44-ve population of iCC603 after 5-FU (38 μg/mL) administration. Similar results were obtained after re-administration of 5-FU (38 μg/mL); however, the rate of the population was reduced. **C** CD44-ve population of iCC603 parent did not proliferate after sorting (*n* = 4). However, the CD44-ve population after 5-FU administration (38 μg/mL) proliferated remarkably. Values are presented as means ± SEM (*P < 0.05, Wilcoxon’s rank-sum test). **D** CD44-ve population from parents and CD44+ve population from DsRed-transfected iCC603 were mixed and administered 5-FU (38 μg/mL). As a result, the surviving cells were composed of DsRed-transfected cells, in which a CD44-ve population appeared. **E** The proliferative potential of CD44-ve and CD44+ve populations after 5-FU administration (38 μg/mL) show that CD44-ve cells started to proliferate slower than CD44+ve cells did; however, the proliferation rate of the CD44-ve population was higher than of the CD44+ve population (*n* = 4). Values are presented as means ± SEM (*P < 0.05, Wilcoxon’s rank-sum test). **F** Subcutaneous organoids were created in mice with both parent and CD44-ve cells after sorting (*n* = 3). After 21 days, CD44-ve cells showed increased tumor sizes (*P* = 0.013, Wilcoxon’s rank-sum test). **G** Drug sensitivity of the CD44-ve population revealed a decreased susceptibility compared to that of the parent (*n* = 4) (*P* = 0.027, Wilcoxon’s rank-sum test). IC50: The half maximal inhibitory concentration

When 5-FU was administered to human organoids, CD44-ve and CD44+ve populations appeared, and after a time-lapse of 60 days or more, their surface markers reverted to the original population (Fig. 3A). Once the CD44-ve population derived from human organoids after 5-FU administration was sorted and cultured, CD44+ve population gradually emerged and CD44-ve population decreased markedly. When oxaliplatin was administered to human organoids, the CD44-ve population also derived after the administration, and CD44+ve population gradually emerged as well (Supplementary Fig. S1). It seems that a parent-like population was reestablished. When 5-FU was readministered to this population, a CD44-ve population emerged again; however, the population rate was reduced (Fig. 3B).

### Origin of the CD44-ve Cells

Compared with the CD44-ve population of organoids parent, the CD44-ve population after chemotherapeutic agent stimulation proliferated remarkably (Fig. 3C). CD44-ve population from parents and CD44+ve population from DsRed-transfected organoids were mixed and administered with chemotherapeutic agents. As a result, the surviving cells were composed of DsRed cells, in which a CD44-ve population appeared (Fig. 3D). Furthermore, we evaluated the CD44 variants. After chemotherapeutic agents, the CD44-ve population showed CD44v9 expression (Supplementary Fig. S2A). Therefore, we focused on CD44v9 expression, and the Cd44-ve population from parents and CD44v9+ve population from DsRed-transfected organoids were mixed and administered chemotherapeutic agents. Therefore, the surviving cells were composed of DsRed cells, in which a CD44v9 population remained (Supplementary Fig. S2B). These results suggest that the CD44-ve population, the persisting cells after chemotherapeutic agents, was derived from the CD44v9 population.

### Functional Analysis

We evaluated the proliferative potential of CD44-ve and CD44+ve populations after chemotherapeutic agent stimulation. CD44-ve cells started to proliferate slower than CD44+ve cells did; however, the proliferation rate of the CD44-ve population was higher than that of the CD44+ve population (Fig. 3E).

After sorting, subcutaneous organoids were created in mice with both parent and CD44-ve cells. After 21 days, CD44-ve cells showed significantly increased tumor sizes (*P* = 0.013; Fig. 3F). Drug sensitivity of the CD44-ve population revealed decreased susceptibility compared to that of the parent (parent IC_50_ 0.52 μg/mL, CD44-ve IC_50_ 1.84 μg/mL; *P* = 0.027; Fig. 3G).

### Single-cell RNA-seq

**Fig. 4:**
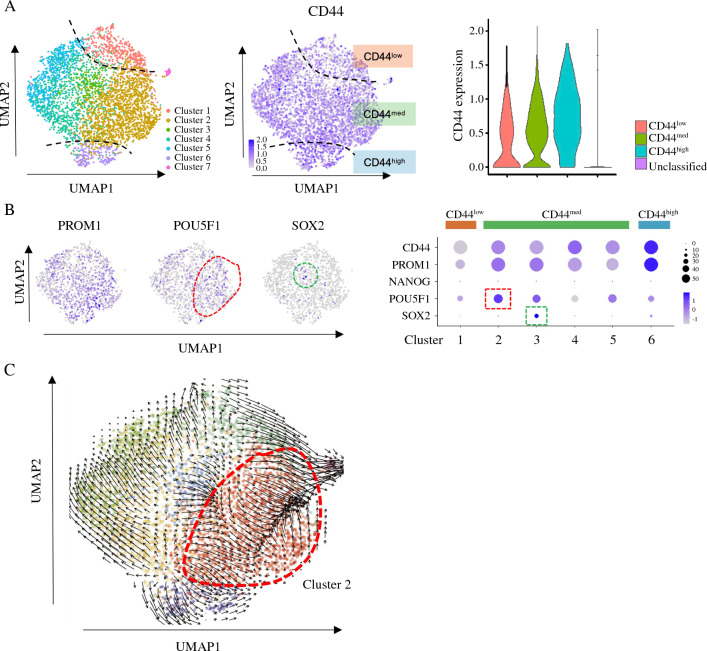
Single-cell RNA-seq. **A** Left: Unsupervised UMAP divided 3,654 cells into seven clusters. Middle: UMAP plot with CD44 expression. Cells were divided into three groups (CD44^low^, CD44^med^, and CD44^high^). Blue and gray indicate high and low expression, respectively. Right: violin plot for CD44. **B** Left: UMAP plots for 3 stem cell markers (*PROM1*, *POU5F1*, and *SOX2*). Right: Dot plot of CD44 and stem cell markers in the 6 clusters. The size of the dots represents the proportion of cells expressing the gene and the color intensity represents the average expression level. **C** The direction of differentiation estimated by RNA velocities is plotted as streamlines on the UMAP

By unsupervised clustering of UMAP, unsorted iCC0603 cells after 5-FU treatment (72 hr) were divided into seven clusters (Fig. 4A left), and these clusters were subcategorized into three groups (CD44^low^, CD44^med^, and CD44^high^), according to the CD44 expression (Fig. 4A middle and right). We estimated that CD44^low^/CD44^med^ population identified by scRNA-seq is identical to that of CD44-ve identified by FC. The expression of stem cell markers (*PROM1*, *NANOG*, *SOX2*, and *POU5F1*) in each subgroup unraveled the heterogeneity of cell population after 5-FU treatment. We found that *POU5F1* was highly expressed in the cluster 2, a subset of CD44^med^ group (Fig. 4B). Furthermore, RNA velocity analysis revealed the dynamic flow into cluster 2, indicating that differentiation into POU5F1-high subset after disposure to chemotherapeutic agents was confirmed at single cell resolution (Fig. 4C).

### Changes in POU5F1 Expression

**Fig. 5:**
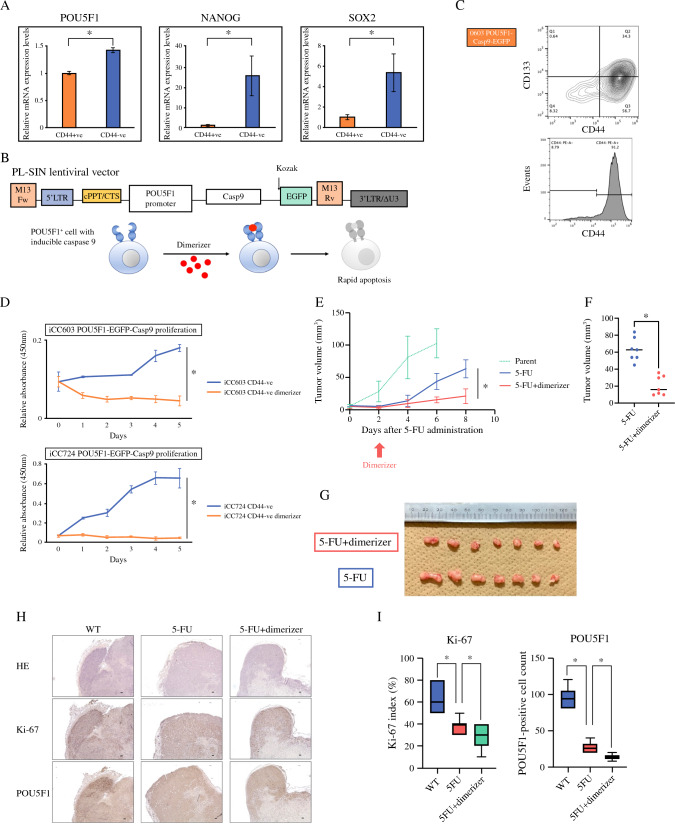
Changes in stem cell-marker expressions. **A** POU5F1, NANOG and SOX2 levels were higher in CD44-ve cells than in CD44+ve cells (*n* = 4). Values are presented as means ± SEM (*P < 0.05, Wilcoxon’s rank-sum test). **B** iCC603 transfected with POU5F1-EGFP-Casp9 vector. **C** POU5F1-EGFP-Casp9 transfected iCC603 resulted in the appearance of a CD44-ve population after 5-fluorouracil (5-FU) administration (38 μg/mL), similar to the organoid iCC603. **D** CD44-ve population of POU5F1-EGFP-Casp9 transfected iCC603 and POU5F1-EGFP-Casp9 transfected iCC724 after 5-FU administration (38 μg/mL) were treated with or without a dimerizer. The CD44-ve cells with a dimerizer did not grow in either primary culture. **E**–**G** Subcutaneous tumors were created in mice with POU5F1-EGFP-Casp9-transfected iCC603 and treated with 5-FU. In the 5-FU group, tumor volume decreased once on Day 2 and then increased. We examined whether the tumor volume changed with or without the dimerizer on Day 2. Subcutaneous tumors of 5-FU treated mice showed a decrease tumor size compared with the tumors without 5-FU administration. In the mice with a single dose of the dimerizer administered on Day 2, the results indicated a significant suppression of tumor growth (*n* = 7) (*P < 0.05, Wilcoxon’s rank-sum test). **H** POU5F1 and Ki-67 of CRC specimens were evaluated in xenograft models. *WT* wild type. Scale bar, 100 µm. **I** The number of Ki-67-positive cells and the percentage of POU5F1-positive cells in subcutaneous tumors with the dimerizer were lower than that without dimerizer (/10HPF). Values are presented as means ± SEM (*P < 0.05, Wilcoxon’s rank-sum test)

POU5F1 expression was examined in CD44-ve/+ cells, in addition to NANOG and SOX2. SOX2, POU5F1, and NANOG levels were higher in CD44-ve cells, indicating the presence of stemness population (Fig. 5A). Organoids transfected with POU5F1-EGFP-Casp9 vector were evaluated (Fig. 5B). POU5F1-EGFP-Casp9 transfected organoids resulted in the appearance of a CD44-ve population after administration of chemotherapeutic agents, similar to the organoid parent (Fig. 5C). CD44-ve population of POU5F1-EGFP-Casp9 transfected organoids after chemotherapeutic agents were treated with or without a dimerizer. The treated CD44-ve cells did not grow in either primary culture (Fig. 5D). Subcutaneous tumors were created in mice with POU5F1-EGFP-Casp9 transfected organoids and treated with chemotherapeutic agents. We examined whether the tumor volume changed with or without the administration of the dimerizer. Subcutaneous tumors of chemotherapeutic agent-treated mice showed a decrease in tumor size compared with those without chemotherapeutic agent administration. The results of the mice with a single dose of the dimerizer administered on Day 2 indicated significant suppression of tumor growth (Figs. 5E–G). The number of Ki-67-positive cells and the percentage of POU5F1-positive cells in subcutaneous tumors in the dimerizer group were lower than that without dimerizer, suggesting that dimerizer treatment suppressed cell proliferation (Figs. 5H, I). Because cell proliferation was suppressed in the dimerizer group, POU5F1 was involved in the treatment resistance.

## Discussion

Unlike cell lines, clinical tissues are heterogeneous populations and can be evaluated in primary cultured cells as a model of tumor heterogeneity (i.e., diversity).^25–27^ Upon chemotherapeutic agent stimulation, CD44-ve cells appeared in organoids but not in the cell lines. These results that newly emerged CD44-ve did not appear in the cell lines suggest that these newly emerged cells depend on the original “diverse” cell population. These unique, emerged cells also had CD44 but not stem cell markers, such as CD24 and CD133. We examined whether the presence of these cells led to resistance to chemotherapeutic agents. CD44 is expressed in many cells and is involved in cell adhesion and migration and in regulating lymphocyte kinetics, such as lymphocyte rolling in immune responses.^15,16,28^ CD44 is known as overexpressed in CRC and has been recognized as a molecular marker of CSCs. CD44 also has several variants that are considered markers of CSCs.^29,30^ Traditionally, CSCs present in the tumor are positive for CD44, which is considered a CRC stem cell marker, and these cells are resistant to chemotherapeutic agents.^31^ It is believed that these stem cells survive and rebuild their original population after chemotherapeutic agent stimulation, exacerbating resistance to chemotherapeutic agents.^32^ CD44+ve cells have been reported to be chemotherapy-resistant as CSCs, but there are no reports on the unique CD44-ve cells that appear transiently after this chemotherapy. Different organoids have different concentrations of CD44-ve appearing. We consider that this is the cause of resistance, because this population appears at higher concentrations and not at lower doses of chemotherapeutic agents. In this study, we hypothesized that CD44-ve cells exist in tumors in addition to conventional CSCs and that these cells, which emerge when the chemotherapeutic agents’ concentration and overall tumor stress increase, are involved in tumor growth and resistance after chemotherapeutic agents. We hypothesized that these cells generate stem cells independently, which would support CSCs when they survive drug administration.

CD44-ve cells reestablish and form a similar population to the original (parental) population, and perhaps, the CD44-ve population contains cells that induce CSCs (i.e., cells that are the source of CSCs). After chemotherapeutic agent stimulation, CD44-ve cells may be derived from CD44+ve cells within the parental line. These results indicate that the emerged CD44-ve cells were derived from CD44+ve cells before chemotherapy, consistent with previous reports.^14,15^ It suggests that the emerged population is the “true” CSCs causing drug resistance. After chemotherapeutic agent stimulation, CD44-ve cells have a higher proliferative capacity and are more malignant than CD44+ve cells. Single-cell analysis showed a higher percentage of cells with POU5F1 (OCT4) expression within the CD44-ve cell population and high stemness after chemotherapeutic agents, such as 5-FU for POU5F1 in vivo. POU5F1 was highly expressed in transient CD44-ve cells, and suppressing the POU5F1 leads to the treatment for the prevention of the recurrence/relapse after chemotherapeutic agent. POU5F1 is likely involved in chemotherapeutic agent resistance to the stemness.

For the treatment against refractory tumors, such as the recurrence after chemotherapeutic agents, the treatment should target the emerging specific population such as CD44 (or CD44v9) as well as proliferative cancer cells.

## Supplementary Information

Below is the link to the electronic supplementary material.

Supplementary file1 (DOCX 122 kb)

## Data Availability

scRNA-seq data can be obtained from the corresponding authors upon reasonable request.
