# Supplementary material for: Cancer Stem Cells Persist Despite Cellular Damage, Emergence of the Refractory Cell Population
Source: Ann Surg Oncol. 2023 Jul 31;30(11):6913–24. doi: 10.1245/s10434-023-13849-x (PMC10506944; doi:10.1245/s10434-023-13849-x)
Supplement: Supplementary file 1 — Supplementary file1 (DOCX 122 kb) [file 10434_2023_13849_MOESM1_ESM.docx]

**Supplementary figures legends**

**Fig. S1 Chemosensitivity assay and flow cytometry in organoids**

**
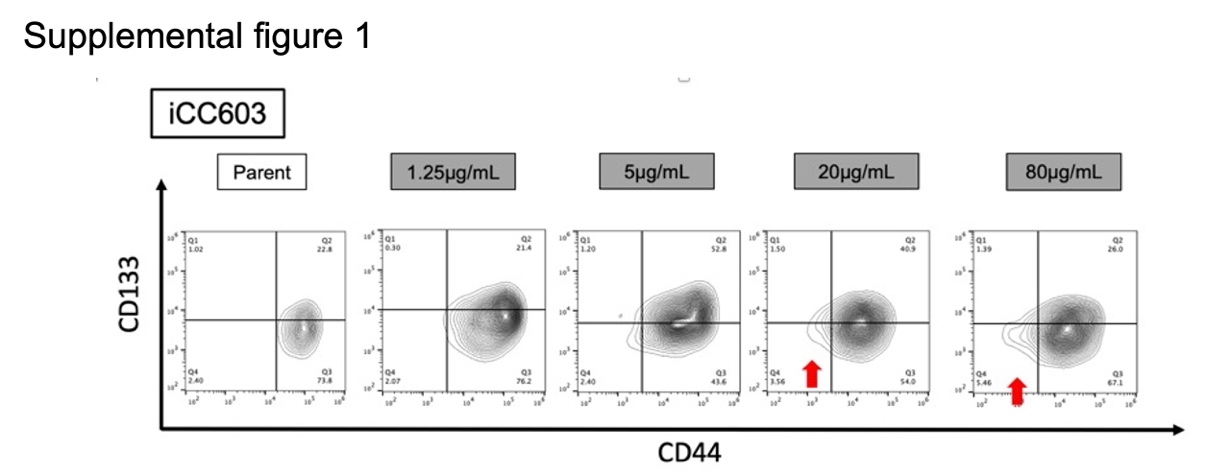
**

Changes in CD44 and CD133 expression after oxaliplatin (80, 20, 5, 1.25 μg/mL) administration to iCC603.

**Fig. S2 CSC markers after the administration of chemotherapeutic agents**

**
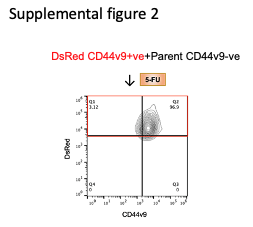
**

CD44v9-ve population from parents and CD44v9+ve population from DsRed-transfected iCC603 were mixed and administered 5-FU (38 μg/mL). As a result, the surviving cells were composed of DsRed cells which are CD44v9+ve population.
